# Interleukin 15 Levels in Serum May Predict a Severe Disease Course in Patients with Early Arthritis

**DOI:** 10.1371/journal.pone.0029492

**Published:** 2011-12-29

**Authors:** Isidoro González-Álvaro, Ana M. Ortiz, José María Alvaro-Gracia, Santos Castañeda, Belen Díaz-Sánchez, Inmaculada Carvajal, J. Alberto García-Vadillo, Alicia Humbría, J. Pedro López-Bote, Esther Patiño, Eva G. Tomero, Esther F. Vicente, Pedro Sabando, Rosario García-Vicuña

**Affiliations:** 1 Rheumatology Service, Hospital Universitario de La Princesa, IIS Princesa, Madrid, Spain; 2 Sanatorio Nuestra Señora del Rosario, Madrid, Spain; University of Southern California, United States of America

## Abstract

**Background:**

Interleukin-15 (IL-15) is thought to be involved in the physiopathological mechanisms of RA and it can be detected in the serum and the synovial fluid of inflamed joints in patients with RA but not in patients with osteoarthritis or other inflammatory joint diseases. Therefore, the objective of this work is to analyse whether serum IL-15 (sIL-15) levels serve as a biomarker of disease severity in patients with early arthritis (EA).

**Methodology and Results:**

Data from 190 patients in an EA register were analysed (77.2% female; median age 53 years; 6-month median disease duration at entry). Clinical and treatment information was recorded systematically, especially the prescription of disease modifying anti-rheumatic drugs. Two multivariate longitudinal analyses were performed with different dependent variables: 1) DAS28 and 2) a variable reflecting intensive treatment. Both included sIL-15 as predictive variable and other variables associated with disease severity, including rheumatoid factor (RF) and anti-cyclic citrullinated peptide antibodies (ACPA). Of the 171 patients (638 visits analysed) completing the follow-up, 71% suffered rheumatoid arthritis and 29% were considered as undifferentiated arthritis. Elevated sIL-15 was detected in 29% of this population and this biomarker did not overlap extensively with RF or ACPA. High sIL-15 levels (β Coefficient [95% confidence interval]: 0.12 [0.06–0.18]; p<0.001) or ACPA (0.34 [0.01–0.67]; p = 0.044) were significantly and independently associated with a higher DAS28 during follow-up, after adjusting for confounding variables such as gender, age and treatment. In addition, those patients with elevated sIL-15 had a significantly higher risk of receiving intensive treatment (RR 1.78, 95% confidence interval 1.18–2.7; p = 0.007).

**Conclusions:**

Patients with EA displaying high baseline sIL-15 suffered a more severe disease and received more intensive treatment. Thus, sIL-15 may be a biomarker for patients that are candidates for early and more intensive treatment.

## Introduction

The optimal strategy to manage rheumatoid arthritis (RA) is currently to start an early and intensive treatment adjusted to a specific target [1], [2], [3], [4], [5]. However, the widespread use of treatment with disease modifying anti-rheumatic drugs (DMARD) in combination may expose some patients with early arthritis (EA) to unjustified risks, while the first line use of biological agents for non-selected patients may be not cost-effective. To overcome these issues, it would be wise to use biomarkers capable of detecting patients at high risk of developing a severe disease. Although rheumatoid factor (RF), anti-citrullinated peptide antibodies (ACPA) and some genetic factors have been associated with an adverse evolution [6], [7], [8], [9], [10], their predictive value is still limited [11]. Therefore, additional markers to predict outcome and therapeutic responses are needed.

Interleukin-15 (IL-15) is thought to be involved in the physiopathological mechanisms of RA. These events include the regulation of cell interactions that promote TNF production [12], [13], [14], and the activation of Th17 lymphocytes driving IL17 production [15], [16], [17], [18]. Through this latter effect, IL-15 regulates the osteoclastogenesis that contributes to juxtaarticular osteoporosis and bone erosion [19], [20], [21], [22]. IL-15 also modulates the functional maturation of dendritic cells and contributes to the survival and activation of neutrophils, B and NK cells [23], [24], [25]. In support of its contribution to RA pathogenesis, IL-15 can be detected in the synovial fluid of inflamed joints in patients with RA but not in patients with osteoarthritis or other inflammatory joint diseases [26], [27], [28]. In fact, IL-15 neutralization improves arthritis in animal models and patients with RA [29], [30].

Unlike synovial fluid, serum samples are commonly used to measure diagnostic and prognostic biomarkers. IL-15 is elevated in the serum of some patients with RA but not in healthy controls [15], [31], [32], [33]. Indeed, we recently showed that measuring serum IL-15 (sIL-15) is a potentially useful biomarker as the elevation of this cytokine in serum is not generalized in patients with EA [34]. Therefore, considering the relevant functions of this cytokine in RA, we aimed to test its utility as a clinical biomarker in our register of patients with EA.

## Methods

### Ethics Statement

The register protocol was reviewed and approved by the Ethics Committee for Clinical Research at the Instituto de Investigación Sanitaria La Princesa. All patients were informed about the study and signed an informed consent form prior to be included in the EA Register.

### Objectives

The hypothesis of this work is that patients with early arthritis and high levels of sIL15 suffer a more severe disease. The specific objectives were to determine whether patients with high sIL15 showed higher disease activity or had greater treatment requirements during their follow-up.

### Participants

All the patients enrolled on our Early Arthritis Clinic (EAC) register between September 2001 and November 2006 were considered in this study. During this period 190 patients were included, although only 171 patients completed the two year follow-up (the last patient ended in November 2008). Data from 638 visits corresponding to these later patients were considered for the analysis.

There were 14 patients lost to follow-up and 5 exitus. Deceased patients were significantly older, had a lower educational level and they also displayed a tendency towards a higher HAQ and DAS28 at baseline than those who finished the follow-up (Table S1). Patients lost to follow-up did not differ significantly from completers (Table S1).

Our EAC covers a population of 500,000 inhabitants, >90% of whom are attended by public health insurance. In addition, all primary care physicians in the area are aware of the EAC. To be referred to the clinic, patients must have two or more swollen joints for at least four weeks and symptoms for less than a year. Patients with other specific causes of arthritis were excluded. Thus, only data from patients that fulfilled the ACR criteria for the diagnosis of RA [35] or with chronic undifferentiated arthritis were analyzed. When the 171 patients that fulfilled the two year follow-up were considered, 71% fulfilled the 1987 criteria for RA classification, while 29% remained as undifferentiated arthritis (UA: Table S2) at the end of the follow-up. These two subpopulations did not differ significantly except that the RA patients had a more severe disease at baseline and the educational level of the UA subpopulation was higher (Table S2).

The register's protocol included four visits during a follow up period of two years (baseline, 6, 12 and 24 months). At each visit, the following data were collected and entered into an electronic database: clinical and demographic information; disease duration at the beginning of the follow up; 28 tender and swollen joint counts (TJC and SJC, respectively); global disease activity on a 100 mm visual analogue scale assessed both by the patient (GDAP) and the physician (GDAPh); Spanish version of the Health Assessment Questionnaire [36]; and laboratory tests including erythrocyte sedimentation rate (ESR), C-reactive protein (CRP) and RF levels assessed by nephelometry (positive>20 UI/ml) and ACPA measured by enzyme immune assay (EIA) (Euro-Diagnostica Immunoscan RA; positive >50 UI/ml).

### Description of procedures

#### 1. Measurement of serum IL-15

sIL-15 was measured using a sandwich EIA as described previously [32], [34]. Cytokine values were calculated from a standard curve and samples that generated values higher than the highest standard were diluted (1∶1) in diluent buffer and assayed again.

Serum samples were measured for IL-15 in a blind manner and the physicians that took the therapeutic decisions were also blind to the sIL-15 concentration during the entire follow-up of the patient. To increase the consistency of the results, samples from each patient were assayed twice, the first time after one year of follow-up (samples from the baseline, six month and twelve month visits) and the second time at the end of the follow-up when all four samples were analysed. The duration of frozen storage at −80°C did not significantly altered the measurement of sIL-15 and, therefore, the mean of the two measurements was considered as the definitive value. The exceptions were the final visit or if there was a variation >30% between the values from any sample. In this latter circumstance, all samples from the patient were re-analysed and the definitive value was the mean of the three measurements.

The results of sIL-15 were then used into two variables: a quantitative variable with the value of the cytokine at each visit in pg/ml and a qualitative variable (yes/no) that referred to whether the baseline value of sIL-15 was >20 pg/ml. This latter value was selected as the threshold since it was previously shown to represent the 90^th^ percentile in a healthy population [34].

The intra-assay coefficient of variation was 18.7±33.1% (mean ± standard deviation) and the inter-assay variability was 32.7±33.7%.

#### 2. Other variables and measurements

Disease activity was assessed by the DAS28 based on the ESR as described previously [37].

Regarding DMARD use, we collected the date of onset and withdrawal to generate a new variable, “Intensity of DMARD treatment” (IDT), which represents the number of days of treatment with each DMARD during the follow-up, adjusted by weighted coefficients as follows:

IDT = [(1× number of days with antimalarials [AM])+(1.5× number of days with methotrexate [MTX], leflunomide [LEF], sulphasalazine, parenteral gold salts or cyclosporin A)+(2× number of days with TNF blockers [aTNF])]/number of days of follow-up.

We also revised the patients' GC prescription, as described previously [38], obtaining the cumulative GC dose in mg of prednisone/month of follow-up.

To determine the risk ratios for high disease activity and IT, we generated two qualitative variables for which the patient was considered to be positive if their mean DAS28 during follow-up or IDT variable were above the 75^th^ percentile of the whole population.

### Statistical analysis

The sample was described in terms of mean and SD of quantitative variables with a gaussian distribution; median and the interquartile range (IQR) if the variables displayed a non-normal distribution and; through an estimate of the proportions for qualitative variables. The Student's t test was applied to compare the means of variables with a normal distribution and the Mann Whitney or Kruskall-Wallis tests were used for variables with a non-normal distribution. A χ^2^ or Fisher's test were used to compare categorical variables.

To determine which factors influenced disease activity during the follow-up, we fitted a population-averaged model by generalized linear models (GLM), nested by patient and visit, using the *xtgee* command of Stata 10.1 for Windows (StataCorp LP, College Station, TX, USA). The only difficulty with using Population Averaged Generalized Estimating Equations (PA-GEE) procedures is in understanding the correlation structure [39]. Since the different working correlation matrices assessed did not differ significantly, we present the data from the exchangeable correlation structure that assessed the largest number of observations. The PA-GEE were first modeled adding all the variables with a *p* value<0.15 in the bivariate analysis. The final models were reached by means of Quasilikelihood under the independence model Information Criterion [40] and Wald tests, removing all variables with *p*>0.05.

Risk ratios for high disease activity or high IDT were estimated through the *binreg* command of Stata using the rr option. This command fits GLM allowing adjustment for confounding factors, and the rr option transforms the β coefficients into risk ratios. We first included all variables with a *p* value<0.15 in the respective bivariate analysis. The final models were reached by means of Bayesian Information Criterion (BIC) removing all variables with *p*>0.05 except those that provided a lower BIC value when they were maintained in the model. The RF, ACPA and sIL-15 were then forced in the model to establish their respective risk ratio for each dependent variable.

## Results

### The elevation of sIL-15 overlaps slightly with the presence of rheumatoid factor or anti-cyclic citrullinated antibodies

As previously described for this population [34], we considered increased levels of sIL-15 when higher than 20 pg/ml and the levels detected for this cytokine ranged from undetectable to 434 pg/ml, being the median level 8.6 pg/ml (Table 1). Patients with increased sIL-15 levels showed a slightly trend to higher disease activity baseline measurements than those with low IL-15 levels, although differences in most of those variables did not reach statistical significance (Table 1).

**Table 1:** Characteristics of the population considering the presence of high levels of IL15.

	Total	IL15 low	IL15 high	p
N (%)	171	121 (70.8)	50 (29.2)	
Female (%)	133 (76.4)	97 (78.9)	36 (70.6)	n.s.
Age at baseline	53 [42–66]	54 [41–67]	51 [43–62]	n.s.
Educational level (%)N – P – S - U	5 – 41 – 31 – 23	6 – 41 – 30 – 23	2 – 41 – 31 – 36	n.s.
Disease duration at baseline (months)	6 [4.2–9]	6.2 [4.5–9.3]	5.6 [3.2–8.6]	n.s.
1987 RA criteria (%)	121 (70.8)	79 (65.3)	42 (84)	0.016
DAS28 at baseline	4.5 [3.3–5.7]	4.4 [3.3–5.8]	4.8 [3.5–5.3]	n.s.
HAQ at baseline	1 [0.5–1.62]	1.1 [0.5–1.625]	0.875 [0.5–1.5]	n.s.
Pain (mm)	48 [24–65]	50 [26–70]	39 [20–60]	0.06
GDA Physician	37 [25–50]	30 [25–50]	41 [25–60]	0.09
RF+(%)	75 (43.9)	49 (39.7)	26 (52)	n.s.
ACPA+(%)	68 (38.6)	43 (35.5)	25 (50)	0.07
IL-15 (pg/ml)	8.6 [2.6–24]	5 [1–9.8]	45 [27–80]	<0.001

Data are shown as the median or percentage. N: number; Educational level N: none; P: primary school; S: secondary school; U: university. DAS28: 28-joint count Disease Activity Score. HAQ: Health Assessment Questionnaire. GDA: global disease assessment. RF: rheumatoid factor. ACPA: anti-cyclic citrullinated peptide antibodies; RA: rheumatoid arthritis.

**Figure 1:**
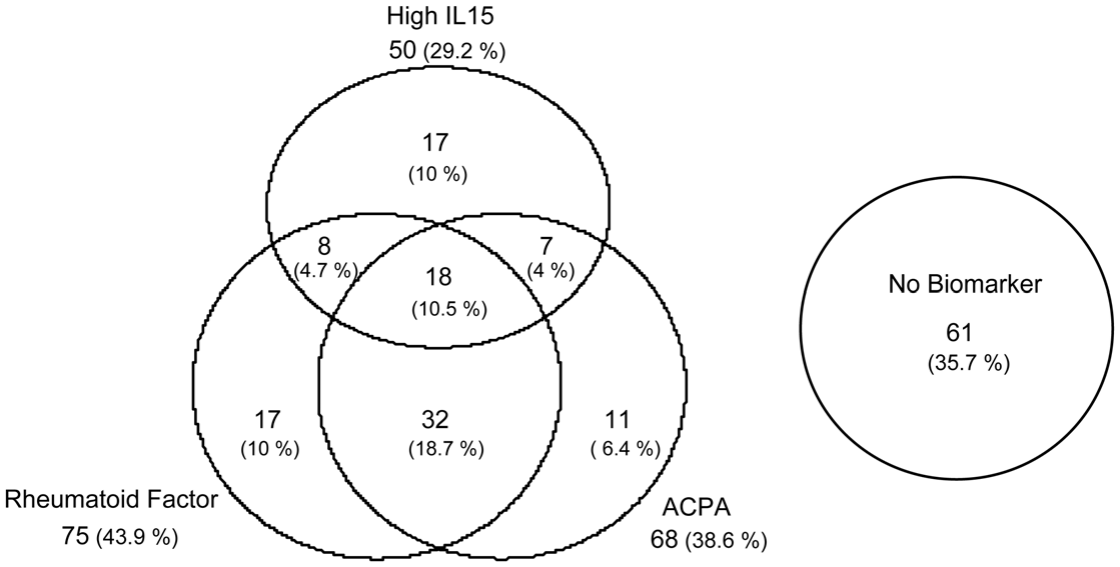
Distribution of rheumatoid factor (RF), anti-cyclic citrullinated peptide antibodies (ACPA) and/or high sIL-15 levels (sIL-15) in the population of patients with Early Arthritis. Data are shown as Venn diagrams whose circle size has been adjusted to represent, albeit not exactly, the number of patients with each combination of markers. The raw number of patients is displayed larger than the percentages that appear in brackets. The total number of patients is 171, although since some patients exhibited combinations of markers, the sum of the number of patients under the name of the biomarkers exceeds this figure.

RF was detected in 43.3% of patients, ACPA in 39.3% and high sIL-15 in 29.2% of the whole population. These three markers were detected more frequently among the patients that fulfilled the RA criteria than in the UA patients (Table S3). On the other hand, none of the markers were detected in 35.7% of the patients and we did not observe an extensive overlap of these markers, except for the presence of both RF and ACPA, especially in patients with RA (Figure 1 and Table S3). These data reflect the heterogeneity among patients with EA.

### Elevated sIL-15 is associated with higher disease activity in patients with EA

**Figure 2:**
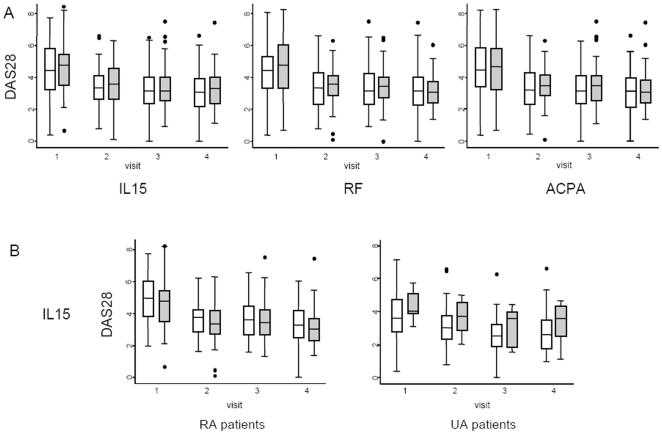
Increased serum IL-15 levels are associated with more severe disease activity during the follow-up of patients with Early Arthritis. A) Evolution of disease activity estimated by DAS28 during the follow-up period in a population of patients with Early Arthritis (EA), and in accordance with the presence of different biomarkers. Left panel: patients with high (grey boxes; n = 50) or low (white boxes; n = 121) sIL-15. Middle panel: patients with positive (grey boxes; n = 75) or negative (white boxes; n = 96) rheumatoid factor reactivity. Right panel: patients with positive (grey boxes; n = 68) or negative (white boxes; n = 103) anti-cyclic citrullinated peptides antibodies. B) Evolution of disease activity estimated by the DAS28 during the follow-up in patients with early Rheumatoid Arthritis (RA; n = 121) or Undifferentiated Arthritis (UA; n = 50) depending on the presence of high (grey boxes) or low (white boxes) IL-15 serum levels. Data are presented as the interquartile range (p75 upper edge of the box, p25 lower edge, p50 midline in the box), as well as the p95 (upper line from the box) and p5. Dots represent the outliers. X-axis shows follow-up visits. Visit 1: baseline; visit 2: six months; visit 3: twelve months; visit 4: twenty four months.

The median disease activity in patients with EA tended to be slightly higher during the follow-up in those with high sIL-15, or with positive RF or ACPA (Figure 2A). However, these differences were not statistically significant. Interestingly, analysis of data from UA subpopulation revealed that patients with elevated sIL-15 at baseline appeared to display clearly higher DAS28 than those with low sIL-15 (Figure 2B). However, due to the small number of patients, these differences were not significant. Moreover, such differences were not observed when patients positive and negative for RF or ACPA were compared (Figure S1).

Since multiple factors may introduce bias when analysing the value of RF, ACPA or sIL-15 as markers of poor prognosis in terms of disease activity, we performed a multivariable longitudinal analysis including sociodemographic and therapeutic variables, as well as these three markers. Accordingly, we found that female gender and advanced age were associated with increased DAS28 scores during the follow-up (Table 2). On the other hand, treatment with MTX, AM, LEF or aTNF were associated with a significant improvement of disease activity in our patients. When adjusted for all these factors, the presence of high baseline sIL-15 was significantly associated with higher DAS28 values during the follow-up (Table 2 model 1). The β coeff. suggests 0.12 increasing DAS28 by each 20 pg/ml increase in baseline sIL-15, then corresponding to 2.4 points in those patients with the highest sIL-15 levels (≈400 pg/ml). Likewise, a positive ACPA but not RF was also associated with higher disease activity. A model without treatments showed a slightly milder yet significant association between sIL-15 and DAS28 values (Table 2, model 2). However, when therapeutic variables were removed from the analysis, ACPA was no longer significantly associated (Table 2, model 2).

**Table 2:** Variables related with the evolution of disease activity in patients with Early Arthritis.

	Model 1		Model 2	
	β Coeff. [95% CI]	p	β Coeff. [95% CI]	P
Female gender	1.0 [0.66–1.46]	<0.001	1 [0.6–1.3]	<0.001
Age at DO (by 10 year)	0.3 [0.2–0.4]	<0.001	0.3 [0.1–0.4]	<0.001
RF Positive	-	n.s.	-	n.s.
ACPA Positive	0.34 [0.01–0.67]	0.044	-	n.s.
IL-15 (by 20 pg/ml)	0.12 [0.06–0.18]	<0.001	0.08 [0.02–0.14]	0.003
Methotrexate	−0.74 [−0.96–−0.52]	<0.001	n.i.	-
Antimalarial	−0.48 [−0.79–−0.18]	0.002	n.i.	-
Sulphasalazine	-	n.s.	n.i.	-
Leflunomide	−0.52 [−0.9–−0.14]	0.007	n.i.	-
Cyclosporine A	-	n.s.	n.i.	-
Gold salts	-	n.s.	n.i.	-
TNF blockers	−1.33 [−2.08–−0.57]	0.001	n.i.	-

Coeff: coefficient; CI: confidence interval; DO: disease onset; RF: rheumatoid factor; ACPA: anti-cyclic citrullinated peptide antibodies; n.s.: not significant; n.i.: not included. Multivariable analysis model 1 includes the effect of DMARD treatment at each visit whereas in model 2, the treatment was not considered.

### High sIL-15 is associated with a greater prescription of DMARDs

The rheumatologists prescribing treatment to the patients were blind to sIL-15 serum values during follow-up but not to their RF or ACPA reactivity. Hence, we examined whether the presence of these markers was associated with more intensive treatment.

The IDT during follow-up was significantly greater in those patients with elevated sIL-15, compared with low sIL-15 (median 1.88 [IQR: 1.5–2.43] vs 1.47 [0.86–1.76]; p<0.001), or with a positive RF (1.6 [1.4–2.27] vs 1.49 [0.82–2.1]; p = 0.024) or positive ACPA (1.61 [1.47–2.17] vs 1.45 [0.57–2.11]; p = 0.034). When the risk ratio for greater IDT was analysed, those patients with high sIL-15 had a significantly higher risk of receiving IDT (RR 2.38, 95% confidence interval 1.54–3.69; p<0.001; Table 3). By contrast, the presence of RF or ACPA was not significantly associated with a higher risk of intensive DMARD treatment (Table 3).

**Table 3:** Risk ratio of Rheumatoid Factor, Anti-cyclic citrullinated peptide antibodies and sIL-15 levels associated with a higher mean disease activity or need for intensive treatment during the follow-up.

	Mean DAS28		Intensive DMARD treatment	
	Risk ratio [95% CI]	p	Risk ratio [95% CI]	p
Rheumatoid Factor +	1.13 [0.63–2.05]	n.s.	1.26 [0.78–2.04]	n.s.
ACPA +	1.57 [0.92–2.67]	n.s.	0.91 [0.56–1.48]	n.s.
sIL-15>20 pg/ml	0.92 [0.66–1.30]	n.s.	2.38 [1.54–3.69]	<0.001
Female gender	2.68 [1.05–6.81]	0.039	-	n.s.
Age at DO: <40 y-o	Ref.	-	-	n.i.
40–55	1.60 [0.47–5.44]	n.s.	-	n.i.
55–70	3.37 [1.07–10.60]	0.038	-	n.i.
>70	6.50 [2.18–19.39]	0.001	-	n.i.

DAS28: 28-joint count Disease Activity Score. DMARD: disease modifying anti-rheumatic drugs. CI: confidence interval. ACPA: anti-cyclic citrullinated peptide antibodies; n.s.: not significant; n.i.: not included. DO: disease onset. Ref.: reference variable.

**Figure 3:**
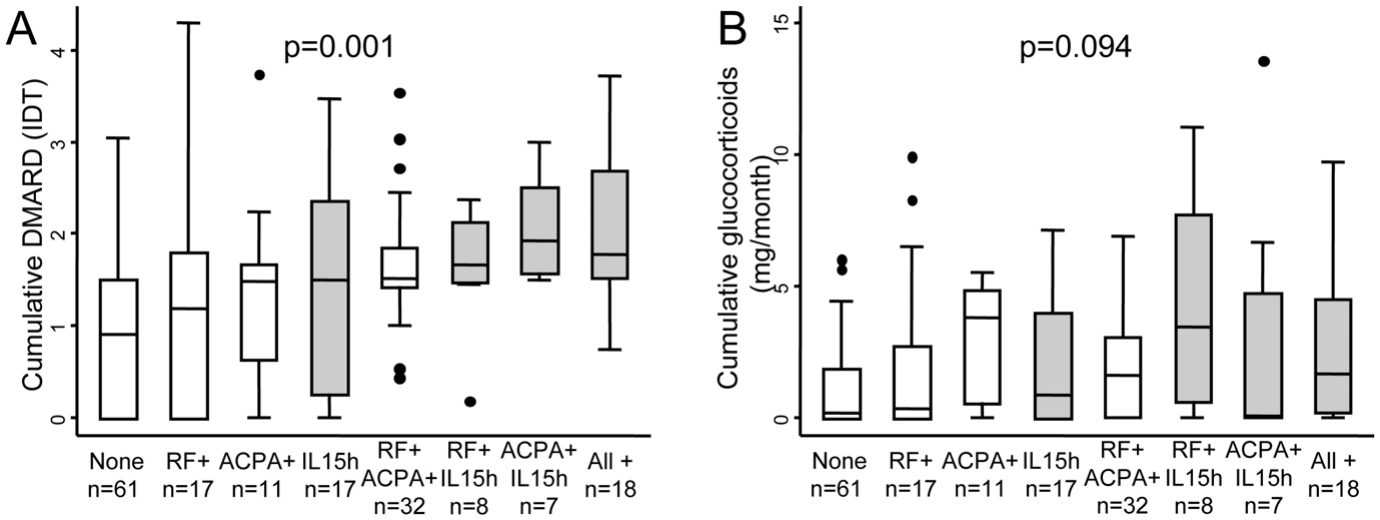
Intensity of treatment in a population of early arthritis patients. A) Cumulative DMARD treatment during the follow-up period, estimated through the IDT variable (see Methods), in the different subpopulations clustered by the elevated IL-15 serum levels (IL-15 h), the presence of rheumatoid factor (RF+) and/or anti-citrullinated peptides antibodies (ACPA+). B) Distribution of the cumulative glucocorticoid dose adjusted to the number of days of follow-up in the different subpopulations clustered by the elevated sIL-15, RF and/or ACPA. Grey boxes represent those patients with high IL-15 alone or in combination with other biomarkers. In all panels the data are presented as the interquartile range (p75 upper edge of the box, p25 lower edge, p50 midline in the box), as well as the p95 (upper line from the box) and p5. Dots represent the outliers. Statistical significance was established through Kruskal-Wallis test.

We then measured the IDT in function of the different combinations of the three markers. Patients with elevated sIL-15 seemed to have a tendency to more intensive DMARD treatment, irrespective of the other markers (Figure 3A, grey boxes; the Kruskall-Wallis' test confirmed a global significant difference among subgroups, while direct paired comparisons with the Mann-Whitney test could not be performed due to the small number of patients in each subgroup).

Regarding the cumulative dose of glucocorticoids, this variable tended to be greater in patients with positive RF, ACPA or high sIL-15, although statistical significance was not reached (Figure 3B and Table S4).

**Figure 4:**
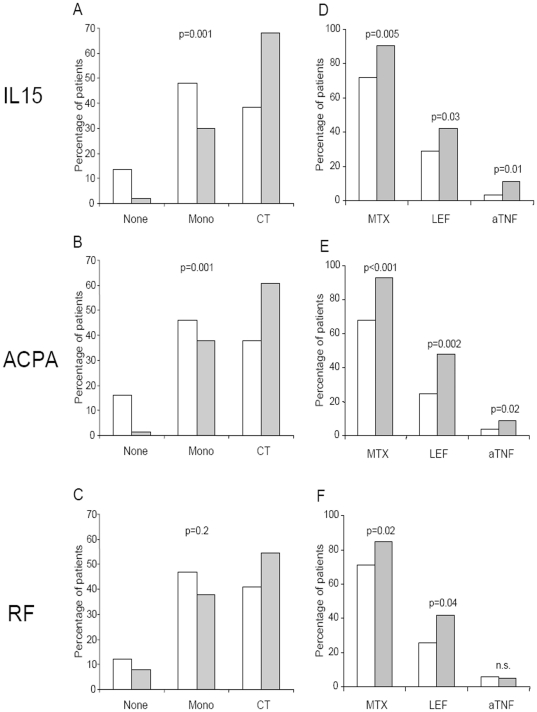
Patterns of DMARD use in a population of early arthritis patients. Left column: Proportion of patients treated with no DMARD (None; n = 17), DMARDs in monotherapy (Mono; n = 75) or in combined therapy (CT; n = 79) in function of the presence (grey columns) or absence (white columns) of high serum IL-15 (A panel), anti-cyclic citrullinated peptide antibodies (ACPA; B panel) or rheumatoid factor (RF; C panel). Statistical significance was established through Fisher's test. Right column: Proportion of patients treated with methotrexate (MTX; n = 133), leflunomide (LEF; n = 57) or tumor necrosis factor blockers (aTNF; n = 10) in function of the presence (grey columns) or absence (white columns) of high serum IL-15 (D panel), anti-cyclic citrullinated peptide antibodies (ACPA; E panel) or rheumatoid factor (RF; F panel). Statistical significance was established through χ^2^ test, except in the case of aTNF use that was analysed through Fisher's test.

A more detailed analysis of DMARD use showed that patients with high sIL15 levels and those with positive ACPA were more frequently prescribed with combined therapy and less frequently managed without DMARDs (Figure 4A and B). However, the presence of RF was not significantly associated with differences in the pattern of use of DMARDs (Figure 4C). In addition, MTX and LEF were more frequently prescribed to patients with high sIL-15, a positive RF or ACPA (Figure 4D to F). Furthermore, the frequency of aTNF prescription was also significantly different when patients were sorted by sIL-15 levels or ACPA (Figure 4D and E), although the number of patients in this situation was low (10 patients), as expected for this type of population. There were no significant differences in the use of antimalarials, sulphasalazine, gold salts or cyclosporin A (data not shown).

## Discussion

Three relevant findings arise from this study: 1) patients with EA and increased sIL-15 were prescribed more intensive DMARD treatment during the two first years of the disease; 2) high levels of IL-15 may be associated with a higher disease activity during the follow-up; and 3) there is heterogeneity in RA regarding the presence of prognosis biomarkers.

This study confirms our previous data reporting the more intensive prescription of DMARDs in those patients with long standing RA and higher sIL-15 [32]; the limitations of that previous study included its retrospective design and the limited number of patients. By contrast, in the current study we included more than 170 patients prospectively followed, in which the use of DMARDs was carefully recorded. Accordingly, we report a clear association between high sIL-15 and the prescription of more intensive treatment, which could be explained in several ways. First, our findings reflect the importance of this cytokine in RA physiopathology [12]–[19], [22] and thus, patients with higher sIL-15 levels may suffer a more severe disease that requires more intensive treatment. Alternatively, sIL-15 could simply be a marker associated to other well known poor prognostic factors in RA. Our data show that there is only partial overlap between sIL-15 and RF or ACPA, which suggests an independent role for sIL-15 as a biomarker. A third explanation could be that the rheumatologists prescribing treatments were biased by other factors. This is unlikely in the case of sIL-15 since physicians were entirely unaware of the value of this parameter in the subjects. By contrast, the association in our study of RF and ACPA with an increased need for DMARDs could have been influenced by the awareness among prescribing rheumatologists of these markers and of their value as poor prognostic factors [6], [7], [9].

In this regard, the association between ACPA or RF reactivity and disease activity during the follow-up was not strong. Indeed, this latter biomarker was not significantly associated with disease activity assessed by DAS28, even when the analysis was adjusted by the treatment prescribed to the patients at each visit. This may be the consequence of the extensive overlap between ACPA antibodies and RF, the former being a better biomarker for disease severity than the latter. By contrast, both IL-15 and ACPA were associated with higher disease activity in an independent manner, and both contributed significantly to our statistical model when it was adjusted to treatment. It should be mentioned, however, that the normal tendency of the members of our unit to obtain a tight control of disease activity is likely to have diminished the ability to estimate the risk ratio for severe disease activity of having elevated sIL-15, positive ACPA or RF reactivity in our population.

Our study also highlights the heterogeneity among patients with EA. Only ACPA and RF overlapped significantly in our population (almost 20% of the total population) and more importantly, >35% of patients did not display any of the biomarkers studied here and some of them had a strong need for treatment. This percentage increases by 10% if IL-15 is not measured, since this is the proportion of patients in our population with increased sIL-15 but no other biomarker. Therefore, further efforts will be necessary to validate new biomarkers, and new candidates may be generated by “-omics” studies (genomic, transcriptomic, proteomic, metabolomic,…) that may help detect patients that need intensive treatment promptly.

Another putative marker for bad prognosis in RA is the presence of increased levels of acute phase reactants at disease onset. The disadvantage of such biomarkers (ESR, CRP or even IL6) is that many patients can be under treatment with glucocorticoids or DMARDs when first evaluated by the rheumatologist. However, we previously found that sIL-15 levels do not change with treatment [34], such that sIL-15 could be used in both naïve and treated patients. In this regard, patients with high sIL-15 displayed increased ESR, CRP and IL6 values at their first visit, although no differences were observed at following visits (Figure S2).

Among the potential limitations of this study we can mention: first, the absence of pre-established treatment strategy in our unit, such as in the BeSt study [1], although data were exhaustively and rigorously collected regarding DMARD treatment, including biological agents. The rheumatologists involved in the study largely follow the treatment recommendations of the Spanish Society of Rheumatology for RA [5], [41], considered especially pro-active in a comparative study of RA management in different European countries [3]. Another shortcoming is that information about RF and ACPA reactivity was only collected as qualitative variables; It is likely that the performance of ACPA, and especially RF, would improve if they were managed as quantitative variables. However, these biomarkers have been studied in depth elsewhere and we wanted to focus on IL-15.

In summary, our data show that patients with EA and high sIL-15 levels at baseline experience a worse disease evolution, despite receiving more intensive treatment. If this finding were reproduced in other populations of patients with EA, sIL-15 levels could serve as a reliable biomarker, alone or in combination with others, to determine which patients are candidates for more intense treatment.

## Supporting Information

Figure S1 **Evolution of disease activity estimated by the DAS28 during the follow-up in patients with early arthritis (EA) depending on the presence of positive (gray boxes) or negative (white boxes) Rheumatoid Factor (RF; Upper panels) or anti-citrullinated peptide antibodies (ACPA; Lower panels).** Left panels: patients that fulfilled Rheumatoid Arthritis criteria during the follow-up. Right panels: patients that remain as Undifferentiated Arthritis at the end of the follow-up. The data are presented as the interquartile range (p75 upper edge of the box, p25 lower edge, p50 midline in the box), as well as the p95 (upper line from the box) and p5. The dots represent the outliers.
(TIF)

Figure S2 **Evolution of interleukin 6 (IL-6) serum levels, erythrocyte sedimentation rate (ESR), C-reactive protein (CRP) and swollen joint count (SJC) in patients with early arthritis depending on the presence of high levels of IL-15 (gray boxes) or low levels of IL-15 (white boxes).** The data are presented as the interquartile range (p75 upper edge of the box, p25 lower edge, p50 midline in the box), as well as the p95 (upper line from the box) and p5. The dots represent the outliers. * p<0.05 Mann-Whitney test.
(TIF)

Table S1 **Characteristics of the patients described in the study and those who did not complete the follow-up.** F-U: follow-up; n: number; IQR: interquartile range; N: none; P: primary school; S: secondary school; U: university; Sp: Spanish; SA: South American; EE: Eastern European; RA: rheumatoid arthritis; UA: undifferentiated arthritis; ACPA: anti-citrullinated peptide antibodies; PhGDA: physician global disease assessment.
(DOC)

Table S2 **Characteristics of the population clustered by final diagnosis.** Data are shown as median or percentage. N: number; IQR: interquartile range. Study level N: none; P: primary school; S: secondary school; U: university. Native country S: Spanish; SA: South American; EE: Eastern Europe. DAS28: 28-joint count Disease Activity Score. HAQ: Health Assessment Questionnaire. GDA: global disease assessment. RF: rheumatoid factor. ACPA: anti-citrullinated peptide antibodies; RA: rheumatoid arthritis; UA: undifferentiated arthritis.
(DOC)

Table S3 **Distribution of rheumatoid factor, anti-cyclic citrullinated peptide antibodies and high levels of IL-15 in patients with Early Arthritis.** RF: rheumatoid factor; ACPA: anti-citrullinated peptide antibodies; Data are shown as the number of patients and the percentage (%). Statistical analyses were performed using the Fisher's test.
(DOC)

Table S4 **Use of glucocorticoids in the population of early arthritis patients, and the subpopulation positive for rheumatoid factor, anti-cyclic citrullinated peptide antibodies or with high serum IL-15 levels.** RF: rheumatoid factor; ACPA: anti-citrullinated peptides antibodies. Statistical analyses were performed using the Kruskal-Wallis's test.
(DOC)

## References

[1] Allaart CF, Breedveld FC, Dijkmans BA (2007). Treatment of recent-onset rheumatoid arthritis: lessons from the BeSt study.. J Rheumatol.

[2] Castro-Rueda H, Kavanaugh A (2008). Biologic therapy for early rheumatoid arthritis: the latest evidence.. Curr Opin Rheumatol.

[3] Emery P, Van Vollenhoven R, Ostergaard M, Choy E, Combe B (2009). Guidelines for initiation of anti-tumour necrosis factor therapy in rheumatoid arthritis: similarities and differences across Europe.. Ann Rheum Dis.

[4] Schoels M, Knevel R, Aletaha D, Bijlsma JW, Breedveld FC (2010). Evidence for treating rheumatoid arthritis to target: results of a systematic literature search.. Ann Rheum Dis.

[5] Tornero Molina J, Sanmartí Sala R, Rodriguez Valverde V, Martín Mola E, Marenco de la Fuente JL (2010). Actualización del Documento Consenso de la Sociedad Española de Reumatología sobre el uso de terapias biológicas en la artritis reumatoide.. Reumatol Clin.

[6] de Vries-Bouwstra JK, Goekoop-Ruiterman YP, Verpoort KN, Schreuder GM, Ewals JA (2008). Progression of joint damage in early rheumatoid arthritis: association with HLA-DRB1, rheumatoid factor, and anti-citrullinated protein antibodies in relation to different treatment strategies.. Arthritis Rheum.

[7] Raza K, Filer A (2009). Predicting the development of RA in patients with early undifferentiated arthritis.. Best Pract Res Clin Rheumatol.

[8] Gonzalez-Gay MA, Gonzalez-Juanatey C, Lopez-Diaz MJ, Pineiro A, Garcia-Porrua C (2007). HLA-DRB1 and persistent chronic inflammation contribute to cardiovascular events and cardiovascular mortality in patients with rheumatoid arthritis.. Arthritis Rheum.

[9] Szodoray P, Szabo Z, Kapitany A, Gyetvai A, Lakos G (2010). Anti-citrullinated protein/peptide autoantibodies in association with genetic and environmental factors as indicators of disease outcome in rheumatoid arthritis.. Autoimmun Rev.

[10] Haupl T, Stuhlmuller B, Grutzkau A, Radbruch A, Burmester GR (2010). Does gene expression analysis inform us in rheumatoid arthritis?. Ann Rheum Dis.

[11] Smolen JS, Aletaha D, Grisar J, Redlich K, Steiner G (2008). The need for prognosticators in rheumatoid arthritis. Biological and clinical markers: where are we now?. Arthritis Res Ther.

[12] Gonzalez-Alvaro I, Dominguez-Jimenez C, Ortiz AM, Nunez-Gonzalez V, Roda-Navarro P (2006). Interleukin-15 and interferon-gamma participate in the cross-talk between natural killer and monocytic cells required for tumour necrosis factor production.. Arthritis Res Ther.

[13] McInnes IB, Leung BP, Sturrock RD, Field M, Liew FY (1997). Interleukin-15 mediates T cell-dependent regulation of tumor necrosis factor-alpha production in rheumatoid arthritis.. Nat Med.

[14] Miranda-Carus ME, Balsa A, Benito-Miguel M, Perez de Ayala C, Martin-Mola E (2004). IL-15 and the initiation of cell contact-dependent synovial fibroblast-T lymphocyte cross-talk in rheumatoid arthritis: effect of methotrexate.. J Immunol.

[15] Ziolkowska M, Koc A, Luszczykiewicz G, Ksiezopolska-Pietrzak K, Klimczak E (2000). High levels of IL-17 in rheumatoid arthritis patients: IL-15 triggers in vitro IL-17 production via cyclosporin A-sensitive mechanism.. J Immunol.

[16] Yoshihara K, Yamada H, Hori A, Yajima T, Kubo C (2007). IL-15 exacerbates collagen-induced arthritis with an enhanced CD4+ T cell response to produce IL-17.. Eur J Immunol.

[17] Cho ML, Ju JH, Kim KW, Moon YM, Lee SY (2007). Cyclosporine A inhibits IL-15-induced IL-17 production in CD4+ T cells via down-regulation of PI3K/Akt and NF-kappaB.. Immunol Lett.

[18] Gonzalez-Alvaro I, Ortiz Garcia AM, Dominguez-Jimenez C, Aragon-Bodi A, Diaz-Sanchez B (2009). Inhibition of TNF and IL-17 production by leflunomide involves the JAK/STAT pathway.. Ann Rheum Dis.

[19] Neumann E, Gay S, Muller-Ladner U (2005). The RANK/RANKL/osteoprotegerin system in rheumatoid arthritis: new insights from animal models.. Arthritis Rheum.

[20] Ogata Y, Kukita A, Kukita T, Komine M, Miyahara A (1999). A novel role of IL-15 in the development of osteoclasts: inability to replace its activity with IL-2.. J Immunol.

[21] Schett G, Hayer S, Zwerina J, Redlich K, Smolen JS (2005). Mechanisms of Disease: the link between RANKL and arthritic bone disease.. Nat Clin Pract Rheumatol.

[22] Miranda-Carus ME, Benito-Miguel M, Balsa A, Cobo-Ibanez T, Perez de Ayala C (2006). Peripheral blood T lymphocytes from patients with early rheumatoid arthritis express RANKL and interleukin-15 on the cell surface and promote osteoclastogenesis in autologous monocytes.. Arthritis Rheum.

[23] Carson WE, Giri JG, Lindemann MJ, Linett ML, Ahdieh M (1994). Interleukin (IL) 15 is a novel cytokine that activates human natural killer cells via components of the IL-2 receptor.. J Exp Med.

[24] Liu CC, Perussia B, Young JD (2000). The emerging role of IL-15 in NK-cell development.. Immunol Today.

[25] Carroll HP, Paunovic V, Gadina M (2008). Signalling, inflammation and arthritis: Crossed signals: the role of interleukin-15 and -18 in autoimmunity.. Rheumatology (Oxford).

[26] McInnes IB, al-Mughales J, Field M, Leung BP, Huang FP (1996). The role of interleukin-15 in T-cell migration and activation in rheumatoid arthritis.. Nat Med.

[27] Ortiz AM, Laffon A, Gonzalez-Alvaro I (2002). CD69 expression on lymphocytes and interleukin-15 levels in synovial fluids from different inflammatory arthropathies.. Rheumatol Int.

[28] Thurkow EW, van der Heijden IM, Breedveld FC, Smeets TJ, Daha MR (1997). Increased expression of IL-15 in the synovium of patients with rheumatoid arthritis compared with patients with Yersinia-induced arthritis and osteoarthritis.. J Pathol.

[29] Baslund B, Tvede N, Danneskiold-Samsoe B, Larsson P, Panayi G (2005). Targeting interleukin-15 in patients with rheumatoid arthritis: A proof-of-concept study.. Arthritis Rheum.

[30] Ruchatz H, Leung BP, Wei XQ, McInnes IB, Liew FY (1998). Soluble IL-15 receptor alpha-chain administration prevents murine collagen-induced arthritis: a role for IL-15 in development of antigen- induced immunopathology.. J Immunol.

[31] Cordero OJ, Salgado FJ, Mera-Varela A, Nogueira M (2001). Serum interleukin-12, interleukin-15, soluble CD26, and adenosine deaminase in patients with rheumatoid arthritis.. Rheumatol Int.

[32] Gonzalez-Alvaro I, Ortiz AM, Garcia-Vicuna R, Balsa A, Pascual-Salcedo D (2003). Increased serum levels of interleukin-15 in rheumatoid arthritis with long- term disease.. Clin Exp Rheumatol.

[33] Suzuki J, Morimoto S, Amano H, Tokano Y, Takasaki Y (2001). Serum levels of interleukin 15 in patients with rheumatic diseases.. J Rheumatol.

[34] Lamana A, Ortiz AM, Alvaro-Gracia JM, Diaz-Sanchez B, Novalbos J (2010). Characterization of serum interleukin-15 in healthy volunteers and patients with early arthritis to assess its potential use as a biomarker.. Eur Cytokine Netw.

[35] Arnett FC, Edworthy SM, Bloch DA, McShane DJ, Fries JF (1988). The American Rheumatism Association 1987 revised criteria for the classification of rheumatoid arthritis.. Arthritis Rheum.

[36] Esteve-Vives J, Batlle-Gualda E, Reig A (1993). Spanish version of the Health Assessment Questionnaire: reliability, validity and transcultural equivalency. Grupo para la Adaptacion del HAQ a la Poblacion Espanola.. J Rheumatol.

[37] Prevoo ML, van 't Hof MA, Kuper HH, van Leeuwen MA, van de Putte LB (1995). Modified disease activity scores that include twenty-eight-joint counts. Development and validation in a prospective longitudinal study of patients with rheumatoid arthritis.. Arthritis Rheum.

[38] Ibanez M, Ortiz AM, Castrejon I, Garcia-Vadillo JA, Carvajal I (2010). A rational use of glucocorticoids in patients with early arthritis has a minimal impact on bone mass.. Arthritis Res Ther.

[39] Hardin J, Hilbe J (2007). Generalized linear models and extensions.

[40] Pan W (2001). Model selection in estimating equations.. Biometrics.

[41] Rodríguez-Valverde V, Cáliz Cáliz R, Álvaro-Gracia Álvaro JM, Marenco de la Fuente JL, Mulero-Mendoza J (2006). III Actualización del Consenso de la Sociedad Española de Reumatología sobre terapia biológica en la artritis reumatoide.. Reumatol Clin.

