# Supplementary figures and images for: Interleukin 15 Levels in Serum May Predict a Severe Disease Course in Patients with Early Arthritis
Source: PLoS One. 2011 Dec 29;6(12):e29492. doi: 10.1371/journal.pone.0029492 (PMC3248461; doi:10.1371/journal.pone.0029492)

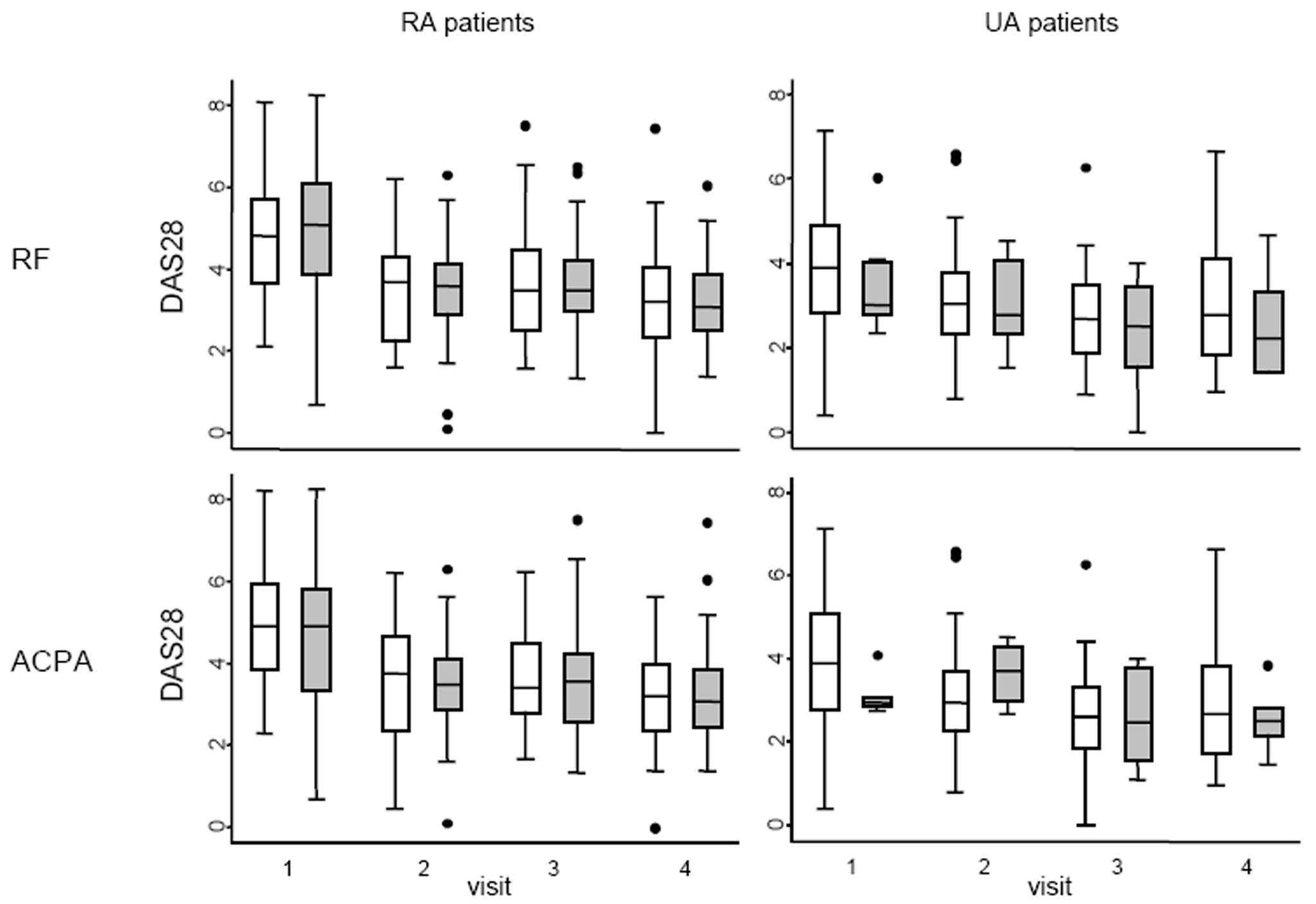

Supplement: Figure S1 — Evolution of disease activity estimated by the DAS28 during the follow-up in patients with early arthritis (EA) depending on the presence of positive (gray boxes) or negative (white boxes) Rheumatoid Factor (RF; Upper panels) or anti-citrullinated peptide antibodies (ACPA; Lower panels). Left panels: patients that fulfilled Rheumatoid Arthritis criteria during the follow-up. Right panels: patients that remain as Undifferentiated Arthritis at the end of the follow-up. The data are presented as the interquartile range (p75 upper edge of the box, p25 lower edge, p50 midline in the box), as well as the p95 (upper line from the box) and p5. The dots represent the outliers. (TIF) [file pone.0029492.s001.tif]

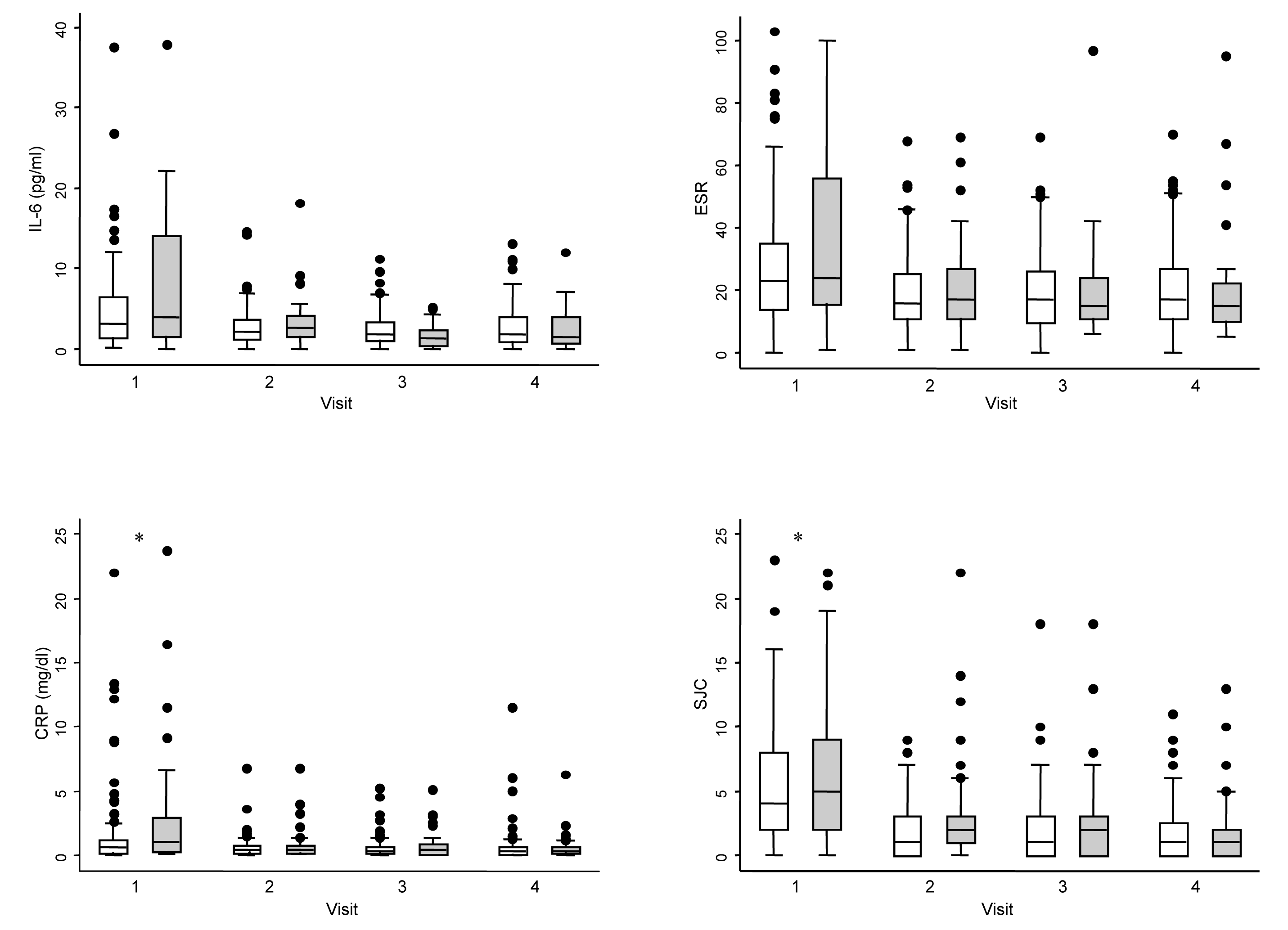

Supplement: Figure S2 — Evolution of interleukin 6 (IL-6) serum levels, erythrocyte sedimentation rate (ESR), C-reactive protein (CRP) and swollen joint count (SJC) in patients with early arthritis depending on the presence of high levels of IL-15 (gray boxes) or low levels of IL-15 (white boxes). The data are presented as the interquartile range (p75 upper edge of the box, p25 lower edge, p50 midline in the box), as well as the p95 (upper line from the box) and p5. The dots represent the outliers. * p<0.05 Mann-Whitney test. (TIF) [file pone.0029492.s002.tif]
