# Supplementary material for: Interleukin 15 Levels in Serum May Predict a Severe Disease Course in Patients with Early Arthritis
Source: PLoS One. 2011 Dec 29;6(12):e29492. doi: 10.1371/journal.pone.0029492 (PMC3248461; doi:10.1371/journal.pone.0029492)
Supplement: Table S1 — Characteristics of the patients described in the study and those who did not complete the follow-up. F-U: follow-up; n: number; IQR: interquartile range; N: none; P: primary school; S: secondary school; U: university; Sp: Spanish; SA: South American; EE: Eastern European; RA: rheumatoid arthritis; UA: undifferentiated arthritis; ACPA: anti-citrullinated peptide antibodies; PhGDA: physician global disease assessment. (DOC) [file pone.0029492.s003.doc]

Table S1. Characteristics of the patients described in the study and those who did not complete the follow-up

|  | Completers | Missed for F-U | Exitus | p |
| --- | --- | --- | --- | --- |
| n | 171 | 14 | 5 |  |
| Female Gender (%) | 77.2 | 78.6 | 40 | 0.15 |
| Age (median[IQR]) | 53 [42 - 66] | 42 [38 - 63] | 77 [75 - 78] | <0.01 |
| Study level  (N – P – S – U; %) | 5 – 41 – 31 – 23 | 0 – 21 – 21 - 58 | 20 – 80 – 0 - 0 | 0.02 |
| Ethnicity  (Sp – SA – EE; %) | 87 – 9 – 4 | 64 – 36 – 0 | 100 – 0 – 0 | 0.07 |
| Diagnosis RA vs UA (%) | 71 – 29 | 50 – 50 | 60 -40 | 0.25 |
| Rheumatoid factor (%) | 43 | 43 | 40 | 0.99 |
| ACPA (%) | 39 | 29 | 25 | 0.63 |
| IL-15 high (%) | 29 | 14 | 20 | 0.45 |
| HAQ at baseline | 1 ± 0.7 | 1.2 ± 0.8 | 1.6 ± 0.7 | 0.28 |
| PhGDA at baseline | 39 ± 25 | 50 ± 21 | 54 ± 18 | 0.28 |
| Pain at baseline | 47 ± 26 | 44 ± 27 | 56 ± 17 | 0.6 |
| DAS28-ESR at baseline | 4.4 ± 1.5 | 5 ± 1.6 | 5.9 ± 0.4 | 0.12 |
| DAS28-CRP at baseline | 4 ± 1.4 | 4.2 ± 1.3 | 5.4 ± 0.5 | 0.14 |

F-U: follow-up; n: number; IQR: interquartile range; N: none; P: primary school; S: secondary school; U: university; Sp: Spanish; SA: South American; EE: Eastern European; RA: rheumatoid arthritis; UA: undifferentiated arthritis; ACPA: anti-citrullinated peptide antibodies; PhGDA: physician global disease assessment.
