# Supplementary material for: Interleukin 15 Levels in Serum May Predict a Severe Disease Course in Patients with Early Arthritis
Source: PLoS One. 2011 Dec 29;6(12):e29492. doi: 10.1371/journal.pone.0029492 (PMC3248461; doi:10.1371/journal.pone.0029492)
Supplement: Table S2 — Characteristics of the population clustered by final diagnosis. Data are shown as median or percentage. N: number; IQR: interquartile range. Study level N: none; P: primary school; S: secondary school; U: university. Native country S: Spanish; SA: South American; EE: Eastern Europe. DAS28: 28-joint count Disease Activity Score. HAQ: Health Assessment Questionnaire. GDA: global disease assessment. RF: rheumatoid factor. ACPA: anti-citrullinated peptide antibodies; RA: rheumatoid arthritis; UA: undifferentiated arthritis. (DOC) [file pone.0029492.s004.doc]

Table S2. Characteristics of the population clustered by final diagnosis

|  | **Total** | **RA** | **UA** | p |
| --- | --- | --- | --- | --- |
| N | 171 | 121 | 50 |  |
| Female (%) | 132 (77.2) | 95 (78.5) | 37 (74) | n.s. |
| Age at baseline | 53 [42 – 66] | 53 [43 – 67] | 52 [35 – 64] | n.s. |
| Study level (%)  N – P – S - U | 5 – 41 – 31 – 23 | 3 – 47 – 32 – 18 | 10 – 24 – 30 – 36 | 0.005 |
| Native country (%)  S – SA – EE | 87 – 9 – 4 | 86 – 9 – 5 | 90 – 8 – 2 | n.s. |
| Disease duration at baseline (months) | 6 [4.2 – 9] | 6 [ 4.2 – 8.5] | 6.4 [4.1 – 10.1] | n.s. |
| DAS28 at baseline | 4.5 [3.3 – 5.6] | 4.8 [3.6 - 5.9] | 3.7 [2.8 – 4.7] | 0.001 |
| HAQ at baseline | 1 [0.5 – 1.62] | 1.12 [0.5 – 1.64] | 0.75 [0.37 – 1.37] | 0.04 |
| Pain (mm) | 48 [24 – 65] | 50 [25 – 66] | 47 [22 – 70] | n.s. |
| GDA Physician | 37 [25 – 50] | 38 [25 – 60] | 25 [14 – 50] | 0.004 |
| RF + (%) | 44 | 53 | 20 | <0.001 |
| ACPA + (%) | 39 | 50 | 12 | <0.001 |
| IL-15 high (%) | 29 | 35 | 16 | 0.014 |

Data are shown as median or percentage. N: number; IQR: interquartile range. Study level N: none; P: primary school; S: secondary school; U: university. Native country S: Spanish; SA: South American; EE: Eastern Europe. DAS28: 28-joint count Disease Activity Score. HAQ: Health Assessment Questionnaire. GDA: global disease assessment. RF: rheumatoid factor. ACPA: anti-citrullinated peptide antibodies; RA: rheumatoid arthritis; UA: undifferentiated arthritis.
