# Supplementary material for: Interleukin 15 Levels in Serum May Predict a Severe Disease Course in Patients with Early Arthritis
Source: PLoS One. 2011 Dec 29;6(12):e29492. doi: 10.1371/journal.pone.0029492 (PMC3248461; doi:10.1371/journal.pone.0029492)
Supplement: Table S3 — Distribution of rheumatoid factor, anti-cyclic citrullinated peptide antibodies and high levels of IL-15 in patients with Early Arthritis. RF: rheumatoid factor; ACPA: anti-citrullinated peptide antibodies; Data are shown as the number of patients and the percentage (%). Statistical analyses were performed using the Fisher's test. (DOC) [file pone.0029492.s005.doc]

Table S3. Distribution of rheumatoid factor, anti-cyclic citrullinated peptide antibodies and high levels of IL-15 in patients with Early Arthritis.

|  | Total  n=171 | RA  n=121 | UA  n= 50 | p |
| --- | --- | --- | --- | --- |
| None | 61 (35.7) | 30 (24.8) | 31 (62) | <0.001 |
| RF | 17 (10) | 11 (9) | 6 (12) |
| ACPA | 11 (6.4) | 8 (6.6) | 3 (6) |
| IL-15 high | 17 (10) | 11 (9) | 6 (12) |
| RF + ACPA | 32 (18.7) | 30 (24.8) | 2 (4) |
| RF + IL-15 high | 8 (4.7) | 7 (5.8) | 1 (2) |
| ACPA + IL-15 high | 7 (4) | 7 (5.8) | 0 |
| RF + ACPA + IL-15 | 18 (10.5) | 17 (14) | 1 (2) |

RF: rheumatoid factor; ACPA: anti-citrullinated peptide antibodies; Data are shown as the number of patients and the percentage (%). Statistical analyses were performed using the Fisher´s test.
