# Supplementary material for: Interleukin 15 Levels in Serum May Predict a Severe Disease Course in Patients with Early Arthritis
Source: PLoS One. 2011 Dec 29;6(12):e29492. doi: 10.1371/journal.pone.0029492 (PMC3248461; doi:10.1371/journal.pone.0029492)
Supplement: Table S4 — Use of glucocorticoids in the population of early arthritis patients, and the subpopulation positive for rheumatoid factor, anti-cyclic citrullinated peptide antibodies or with high serum IL-15 levels. RF: rheumatoid factor; ACPA: anti-citrullinated peptides antibodies. Statistical analyses were performed using the Kruskal-Wallis's test. (DOC) [file pone.0029492.s006.doc]

Table S4. Use of glucocorticoids in the population of early arthritis patients, and the subpopulation positive for rheumatoid factor, anti-cyclic citrullinated peptide antibodies or with high serum IL-15 levels.

|  | Whole population | RF positive | p | ACPA positive | p | high IL-15 | p |
| --- | --- | --- | --- | --- | --- | --- | --- |
| Percentage of patients | 71 | 76 | 0.17 | 78 | 0.1 | 77 | 0.31 |
| Cumulative prednisone dose (mg/month) | 31 [0 – 77] | 40 [3 – 87] | 0.06 | 41 [1 – 90] | 0.09 | 34 [1 – 134] | 0.12 |

RF: rheumatoid factor; ACPA: anti-citrullinated peptides antibodies. Statistical analyses were performed using the Kruskal-Wallis´s test.
